# Editorial: Genome-wide association studies of COVID-19 among diverse human populations

**DOI:** 10.3389/fgene.2022.1088026

**Published:** 2022-12-01

**Authors:** Zhong-Shan Cheng

**Affiliations:** St. Jude Children’s Research Hospital, Memphis, TN, United States

**Keywords:** COVID-19, long COVID, GWAS, SNP, genetic association, sex-biased, African

It is my pleasure to share with research communities this Research Topic on genome-wide association studies (GWAS) relating to coronavirus disease 2019 (COVID-19), titled “*Genome-wide association studies of COVID-19 among diverse human populations*.” Our effort to identify COVID-19 risk factors that are relevant to individuals with a variety of different ancestries is a fundamentally important endeavor, and such findings can be generated by mining open-source data consisting of GWAS summary statistics shared by the GRASP COVID-19 portal (Thibord et al., 2022) and the COVID-19 Host Genetic Initiative (HGI) (Velavan et al., 2021).

The COVID-19 pandemic was caused by the severe acute respiratory syndrome coronavirus 2 (SARS-CoV-2) (Hu et al., 2021), which has devastated global populations during the past 3 years. Up to this point, the global death toll caused by COVID-19 is ∼6.6 million according to the COVID-19 dashboard (Dong et al., 2020). Our knowledge of SARS-CoV-2 infection has increased at the price of a heavy fatality rate over the past 3 years. We now know that SARS-CoV-2 exploits human host factors to infect target cells by utilizing its viral spike (S) protein to bind human ACE2 protein on the surface of cells, and subsequent cleavage of the S protein by the human serine protease TMPRSS2 primes the infection by allowing fusion of the viral and lysosomal membranes (Hoffmann et al., 2020). COVID-19 induced by SARS-CoV-2 infection varies in symptoms, ranging from asymptomatic or mild COVID-19 to COVID-19 that may even be life-threatening; this manifests in the form of acute respiratory distress or excessive inflammation, which are hallmarks of severe COVID-19 (Wong et al., 2004; Zhang et al., 2020). An increasing number of studies also suggest that 5%–50% of COVID-19 patients later experience long COVID (Ledford, 2022), this being a more complex condition with over 200 symptoms, including “brain fog,” fever, fatigue, exhaustion, heart damage, and other chronic conditions such as depression and cognitive impairments that last for months or even years after SARS-CoV-2 infection. The exact cause of long COVID is still under investigation, with one leading theory pointing to the persistence of fragments of SARS-CoV-2 virus in the tissue of long COVID patients, causing prolonged symptoms (Yang et al., 2021), and other hypotheses suggesting that SARS-CoV-2 infection triggers a shift in the balance of the renin–angiotensin system (RAS) from the Mas (angiotensin-converting enzyme 2 [ACE2]/angiotensin [Ang] 1–7/Mas) axis to the RAS (Ang-converting enzyme [ACE]/Ang II/Ang II type I receptor [AT1R]) axis (Uysal et al., 2022). A new study has further revealed that the development of autoantibodies against Ang II in severe COVID-19 is correlated with blood pressure dysregulation and COVID-19 severity (Briquez et al., 2022).

Genetic factors have been found to contribute to variation in COVID -19 symptoms; although rapidly evolving and mutating SASR-CoV-2 strains may also lead to different COVID-19 symptoms, the effect of this variation on COVID-19 severity may not be comparable to the major effect contributed by host genetic factors. For example, in the Research Topic, Gjorgjievska et al. report on two cases in which two SARS-CoV-2 variants, namely Omicron BA.1 and BA.2, were detected in a single individual, and the Omicron BA.2 variant subsequently became the dominant one in both these two patients. However, their symptoms were mild, with no symptoms after 4 days. In further support of the above view, a promoter SNP of the SARS-CoV-2 receptor ACE2 has been demonstrated by Luo et al. to be associated with both higher levels of *ACE2* expression in brain tissues and lower risk of COVID-19 hospitalization; variation in *ACE2* may impact both severe COVID-19 and long COVID. Aside from genetic elements of the SARS-CoV-2 receptor *ACE2*, other genetic factors associated with predisposition to severe COVID-19 have been identified (Anastassopoulou et al., 2020; Gandhi et al., 2020; Severe Covid-19 GWAS Group, 2020; Pairo-Castineira et al., 2021), such as single nucleotide polymorphisms (SNPs) or indels located in *OAS* genes, *IFNAR2*, *CCR9*, and *CXCR6*, as well as other genes (Pairo-Castineira et al., 2021; Zhou et al., 2021). In the Research Topic, Zecevic et al. detected a SNP close to *KLHL1* that is associated with COVID-19 in the Serbian population. Glessner et al. also identified several genes (*SEMA6D*, *FMN1*, *ACTN1*, *PDS5B*, *NFIA*, *ADGRL3*, *MMP27*, *TENM3*, *SPRY4*, *MNS1*, and *RSU1*) potentially associated with COVID-19 susceptibility in children. Li et al. reported the nominal associations of potential regulatory SNPs mapped to *TNF*, *IFNAR2*, *APOE*, *FOXP4-AS1*, *ABO*, and *IFITM3*, with COVID-19 in Chinese population.

Sex differences in responses to severe COVID-19 and long COVID are increasingly recognized by researchers. During the early pandemic, with no COVID-19 vaccine available to the global population, there was a tendency toward higher rates of COVID-19 hospitalization and death among men. This may be attributed to the differences between sexes in immune responses to SARS-CoV-2 infection, as women produce more robust immune responses to the infection (Takahashi et al., 2020; Shattuck-Heidorn et al., 2021; Takahashi et al., 2021; Danielsen et al., 2022). Nevertheless, women have been reported to have a higher chance of experiencing long COVID (Sylvester et al., 2022). In the Research Topic, Luo et al. performed sex-biased GWAS analysis for COVID-19 hospitalization and identified three immunity genes (*TRIM21*, *TRIM29*, and *PVLR1*) and two brain-related genes (*KNDC1* and *STK32C*) showing sex-biased effects on COVID-19 hospitalization. The expression of the two aforementioned genes related to brain function is interrupted during SARS-CoV-2 infection, strongly suggesting their potential involvement in COVID-19 or long COVID.

Genetic ancestry may also affect COVID-19 severity. Raza and Abbasi report that the COVID-19 risk SNP rs2236757 residing in the *IFNAR2* gene has undergone recent positive selection in the African population. However, most recently published findings on COVID-19 risk SNPs or genes are mainly found in populations of European ancestry; few genetic risk factors for COVID-19 have been identified in other populations, such as African populations. In the Research Topic, Petersen et al. discuss the limited reporting of COVID-19 risk markers among African populations and point out that this limitation could result in health disparities between African populations and other groups, as it is difficult to extrapolate published COVID-19 genetic risk factors from European populations to African or other populations.

Since multiple comorbidities, such as hypertension, diabetes, and lung cancer, have been observed along with severe COVID-19, genetic factors associated with predisposition to both COVID-19 and these comorbidities are reasonable targets for investigation. To investigate the connection between hypertension and severe COVID-19, Cai et al. performed meta-analyses of 68 observational studies related to COVID-19 severity and hypertension; they concluded that hypertension patients with COVID-19 have a 1.8-fold chance of experiencing severe COVID-19. Further genome-wide cross-trait meta-analysis revealed that genetic variants of genes expressed in the lung, such as *CCR1*/*CCR5* and *IL10RB*, may confer liability to both hypertension and severe COVID-19. Additionally, Luo et al. (under peer review) searched for genetic factors affecting the outcomes of both hypertension and COVID-19 and identified one regulatory SNP located in the gene *SPEG* that is potentially associated with severe COVID-19 in women but not men. Furthermore, Faridzadeh et al. report an association between an indel of *ACE1* and severe COVID-19 in the Iranian population.

In summary, the open-source data consisting of GWAS summary statistics shared by GRASP and HGI have dramatically propelled global research communities toward discoveries of genetic risk markers for COVID-19. Long COVID is now emerging among COVID-19 patients and may devastate more patients worldwide. Given the greater complexity of long COVID and the substantial number of patients who may experience this condition, global research communities need to work together to reduce the medical burden imposed by it and ultimately to eliminate it.
